# Visualizing lipid nanoparticle trafficking for mRNA vaccine delivery in non-human primates

**DOI:** 10.1016/j.ymthe.2025.01.008

**Published:** 2025-01-10

**Authors:** Maureen Buckley, Mariluz Araínga, Laura Maiorino, Ivan S. Pires, B.J. Kim, Katarzyna Kaczmarek Michaels, Jonathan Dye, Kashif Qureshi, Yiming J. Zhang, Howard Mak, Jon M. Steichen, William R. Schief, Francois Villinger, Darrell J. Irvine

**Affiliations:** 1 Department of Biological Engineering, Massachusetts Institute of Technology, Cambridge, MA 02139, USA; 2 Koch Institute for Integrative Cancer Research, Massachusetts Institute of Technology, Cambridge, MA 02139, USA; 3 Department of Chemical Engineering, Massachusetts Institute of Technology, Cambridge, MA 02139, USA; 4 Ragon Institute of Massachusetts General Hospital, Massachusetts Institute of Technology, Harvard University, Cambridge, MA 02139, USA; 5 Howard Hughes Medical Institute, Chevy Chase, MD 20815, USA; 6 Department of Materials Science of Engineering, Massachusetts Institute of Technology, Cambridge, MA 02139, USA; 7 New Iberia Research Center, University of Louisiana at Lafayette, Lafayette, LA 70560, USA; 8 Consortium for HIV/AIDS Vaccine Development (CHAVD), The Scripps Research Institute, La Jolla, CA 92037, USA; 9 Department of Immunology and Microbiology, The Scripps Research Institute, La Jolla, CA 92037, USA; 10 IAVI Neutralizing Antibody Center, The Scripps Research Institute, La Jolla, CA 92037, USA

**Keywords:** mRNA vaccines, lipid nanoparticles, vaccine biodistribution, PET-CT imaging, non-human primate

## Abstract

mRNA delivered using lipid nanoparticles (LNPs) has become an important subunit vaccine modality, but mechanisms of action for mRNA vaccines remain incompletely understood. Here, we synthesized a metal chelator-lipid conjugate enabling positron emission tomography (PET) tracer labeling of LNP/mRNA vaccines for quantitative visualization of vaccine trafficking in live mice and non-human primates (NHPs). Following intramuscular injection, we observed LNPs distributing through injected muscle tissue, simultaneous with rapid trafficking to draining lymph nodes (dLNs). Deltoid injection of LNPs mimicking human vaccine administration led to stochastic LNP delivery to three different sets of dLNs. LNP uptake in dLNs was confirmed by histology, and cellular analysis of tissues via flow cytometry identified antigen-presenting cells as the primary immune cell type responsible for early LNP uptake and mRNA translation. These results provide insights into the biodistribution of mRNA vaccines administered at clinically relevant doses, injection volumes, and injection sites in an important large animal model for vaccine development.

## Graphical abstract

**Figure:**
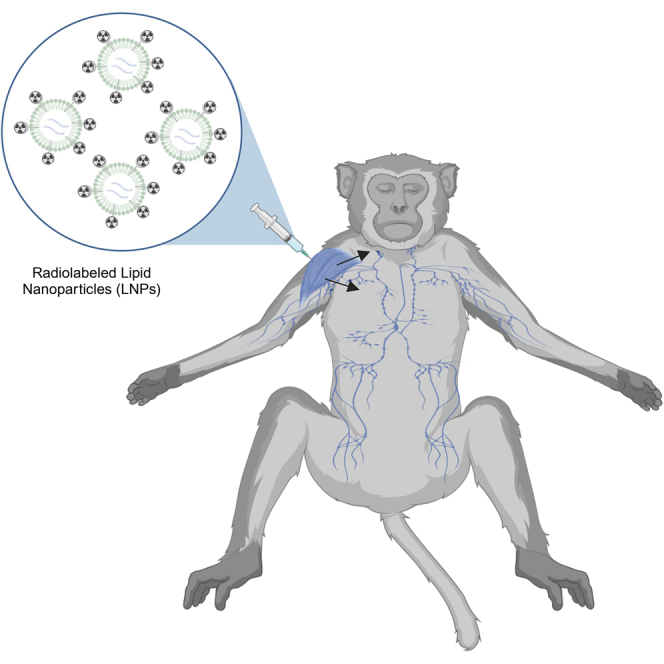

## Teaser

Buckley and colleagues report a strategy for *in situ* imaging of mRNA-lipid nanoparticle vaccines in live animals. Lipid nanoparticles were observed in muscle tissue and draining lymph nodes, with stochastic delivery found in different lymph node sets. Understanding mRNA-lipid nanoparticle biodistribution and trafficking kinetics is crucial for future vaccine development.

## Introduction

Major technological advancements for the use of mRNA as a therapeutic have been made over the past 20 years. Important innovations include the discovery of base modifications to modulate the lifetime and innate immune stimulatory capacity of mRNA, and the development of efficacious delivery vehicles that allow for effective delivery of mRNA *in vivo*.^1^^,^^2^^,^^3^^,^^4^ For application in vaccines, pseudouridine base modifications weaken the recognition of mRNA by innate immune sensors, providing more efficient translation and antigen expression without induction of excess inflammation.^2^^,^^3^ mRNA vaccines provide for a faster synthesis process than traditional protein-based vaccines and are readily manufactured at scale, making them a cost-effective solution for rapid therapeutic development.^1^ During the SARS-COV-2 pandemic, the first Food and Drug Administration-approved mRNA vaccines proved to be highly effective in mitigating the incidence and severity of COVID-19.^5^ Among different types of delivery vehicles, lipid nanoparticles (LNPs) stand as the most clinically advanced, with all currently approved mRNA vaccines utilizing LNPs to deliver their payloads.^1^^,^^4^

Although traditional subunit vaccines are most often administered intramuscularly, primary immune responses are initiated in draining lymph nodes via injected antigen convecting into draining lymph vessels or being taken up by antigen-presenting cells that migrate to dLNs.^6^ While mechanisms of antigen dispersal and the biodistribution behavior of subunit vaccines have been studied in detail,^7^^,^^8^ much less is known about the fate of LNP/mRNA vaccines, especially in large animals and humans. In mice, LNPs have been found to be taken up by conventional dendritic cells (DCs) and infiltrating immune cells in the lymph node, with mRNA vaccine-encoded reporter protein detectable in the subcapsular sinuses of lymph nodes.^9^^,^^10^^,^^11^ In non-human primates (NHPs), mRNA/LNP vaccines were shown to induce strong innate immune activation in draining lymph nodes, with tagged mRNA payload and reporter protein-encoded mRNA detected at the injection site and dLNs following intramuscular (i.m.) injection.^12^^,^^13^ However, in these studies the specific muscle site that was injected was either not identified or multiple muscle injection sites were pooled for analysis, and thus it remains unclear if different anatomic sites (e.g., quadriceps vs. deltoid) exhibit different patterns of mRNA vaccine distribution. Notably, at very early times post-immunization (4 h), neither LNPs nor mRNA-expressed protein were detected in dLNs,^12^ but both LNPs and mRNA payloads were readily measured in dLN antigen-presenting cells (APCs) by 24 h.^12^^,^^13^

From this prior work, it has remained unclear (1) how reliable or stochastic is LNP uptake in different draining lymph node basins in large animals, (2) whether there is a significant contribution of direct drainage of LNPs to lymph nodes (vs. cell-mediated transport of mRNA/LNPs), and (3) how many LNs are accessed following mRNA vaccination using clinically relevant injection volumes/sites. To begin to address these questions, here we carried out studies tracking LNPs and mRNA in mice and NHPs following administration of mRNA vaccines encoding reporter proteins or a stabilized HIV Env immunogen, using LNP compositions mimicking that used in the approved Moderna COVID vaccine. We developed radiometal chelator-tagged LNPs enabling whole-animal positron emission tomography (PET) imaging of the fate of vaccine carriers, and complemented whole-animal imaging of LNP distribution with tissue-level histology, flow cytometry, and qPCR analysis of vaccine distribution in dLN tissues. These studies revealed that following i.m. injection, LNPs were localized in injected muscles, but could also be detected within 4 h in dLNs, with stochasticity in terms of which LNs accumulated vaccine. Combined tissue-level analyses on LNs recovered 40 h post i.m. injection confirmed the presence of LNPs and mRNA in dLNs and revealed APC populations as the primary immune cell types exhibiting LNP uptake and mRNA translation. These findings provide new insights into the biodistribution of mRNA vaccines at the whole-animal level and provide a rationale for understanding the potency of this vaccine modality.

## Results

### Radiometal-chelating LNPs enable loading of PET tracers while retaining mRNA delivery function

**Figure 1:**
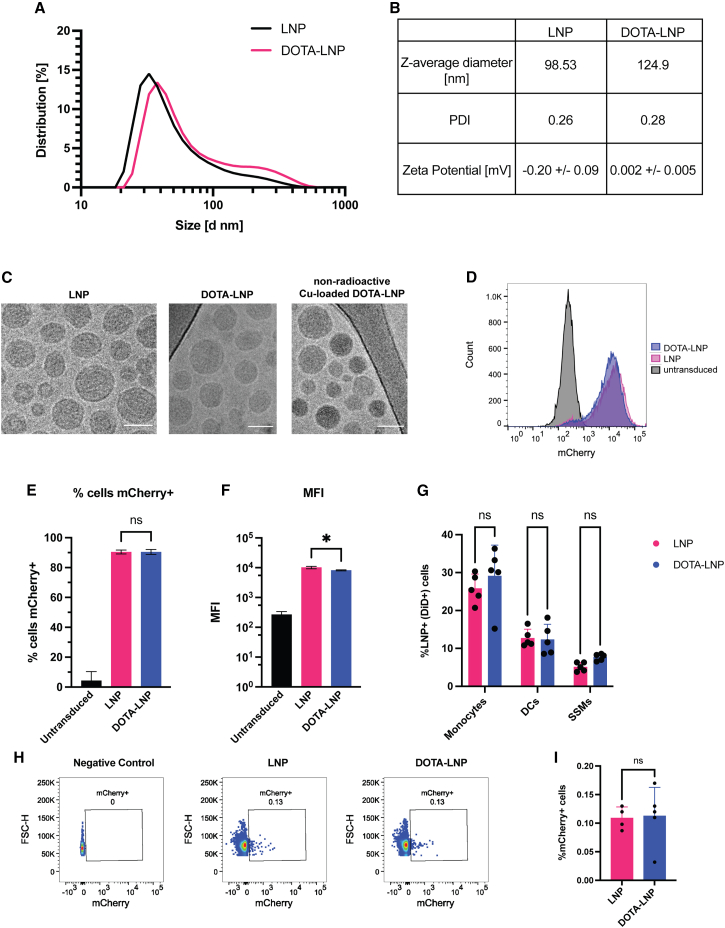
Radiometal-chelating LNPs enable loading of PET tracers while retaining mRNA delivery function (A) Dynamic light scattering (DLS) analysis of LNPs with or without DOTA-lipid. (B) LNP quality control metrics table for LNPs with or without DOTA-lipid. (C) CryoTEM imaging of LNPs, DOTA-LNPs, and non-radioactive Cu-loaded DOTA-LNPs. Scale bars, 50 nm. (D–F) C2C12 cells were incubated with 10 μg/mL mCherry-encoding mRNA delivered by LNPs or non-radioactive Cu-loaded DOTA-LNPs for 24 h, then analyzed by flow cytometry for mCherry expression. Shown are histograms of mCherry fluorescence (D), percentages of mCherry-positive cells (E), and mean fluorescence intensities of transfected cells (F). Statistical significance was determined by one-way ANOVA followed by Tukey’s post hoc test. ∗*p* < 0.05; ∗∗∗∗*p* < 0.0001. All data show means ± SEM. (G) BALB/c mice (*n* = 5 animals/group) were immunized i.m. with 10 μg of LNPs or non-radioactive Cu-loaded DOTA-LNPs that were labeled with DiD. Popliteal LNs were harvested at 12 h and analyzed for the percentage of DiD^+^ cells among monocytes, DCs, and subcapsular sinus macrophages (SSMs). (H and I) BALB/c mice (*n* = 4–5 animals/group) were immunized i.m. with 10 μg LNPs or non-radioactive Cu-loaded DOTA-LNPs encapsulating mCherry mRNA. Popliteal LNs were harvested 24 h post-injection and assessed for mCherry expression by flow cytometry. Shown are representative flow plots of mCherry expression in live cells recovered from negative control lymph nodes, LNP-immunized, and non-radioactive Cu-loaded DOTA-LNP-immunized lymph nodes (H), and the percentages of mCherry-positive cells (I).

To visualize LNP trafficking *in vivo*, we synthesized a lipid functionalized with the metal chelator 1,4,7,10-tetraazacyclododecane-1,4,7,10-tetraacetic acid (DOTA) that could be incorporated into the nanoparticles. Bicyclononyne-DOTA was reacted with aziodethyl phosphatidyl choline to form DSPC-DOTA (Figure S1A). The DOTA conjugate was purified by HPLC, and its identity verified by mass spectrometry (Figures S1B and S1C; Table S1). In parallel, base-modified mRNA encoding either mCherry as a reporter gene or a transmembrane form of the stabilized HIV Env trimer N332-GT2gp151 (note that we will refer to it as N332-GT2 for simplicity)^14^^,^^15^ was prepared by *in vitro* transcription. mRNA-loaded LNPs with compositions mimicking the Moderna COVID-19 mRNA vaccine formulation were prepared incorporating 0.5 mol% of the DSPC-DOTA (DOTA-LNPs). Dynamic light scattering (DLS) showed that LNPs prepared with the DOTA-lipid had slightly larger mean particle size, but similar overall particle size distributions, polydispersities, and zeta potentials as LNPs prepared without the DOTA-lipid (Figures 1A and 1B). Transmission electron cryomicroscopy (cryoTEM) imaging of the LNPs also revealed similar morphologies for the LNPs prepared with or without incorporated DSPC-DOTA (Figure 1C). In addition, we imaged DOTA-LNPs that had been loaded with non-radioactive copper and found the particle size/morphologies to be largely unchanged (Figure 1C). To confirm that inclusion of the chelator lipid did not affect the transfection activity of the LNPs, C2C12 murine myoblast cells were transfected with mCherry mRNA encapsulated in non-tagged LNPs or DOTA-LNPs loaded with ^64^Cu that had been allowed to decay to undetectable levels of radioactivity. Transfection efficiencies and mean fluorescence intensities of mCherry expression were identical between the two groups (Figures 1D–1F). Thus, incorporation of a low level of tagged lipid enables LNPs to be generated that carry a radiometal chelator and are fully functional for mRNA delivery.

In order to validate that LNP biodistribution is not altered by the addition of DOTA, two different animal studies were carried out. First, LNPs and non-radioactive copper-loaded DOTA-LNPs were labeled with the fluorescent lipid DiD and administered i.m. to BALB/c mice. Draining popliteal LNs were harvested 12 h post-immunization and assessed for DiD signal. An identical pattern of particle uptake was found in monocytes, DCs, or subcapsular sinus macrophages for LNPs or DOTA-LNPs (Figure S2; Figure 1G). Next, we immunized BALB/c mice with LNPs or non-radioactive copper-loaded DOTA-LNPs carrying mCherry-encoding mRNA. Popliteal LNs were harvested 24 h post-immunization, and mCherry expression among live cells was found to be essentially identical for LNPs and DOTA-LNPs (Figures 1H and 1I). These results suggest that the inclusion of copper-loaded DSPC-DOTA into the LNP formulation does not have a major impact on LNP trafficking, cellular uptake, or mRNA expression.

### LNPs distribute in injected muscle and rapidly reach draining lymph nodes in mice

**Figure 2:**
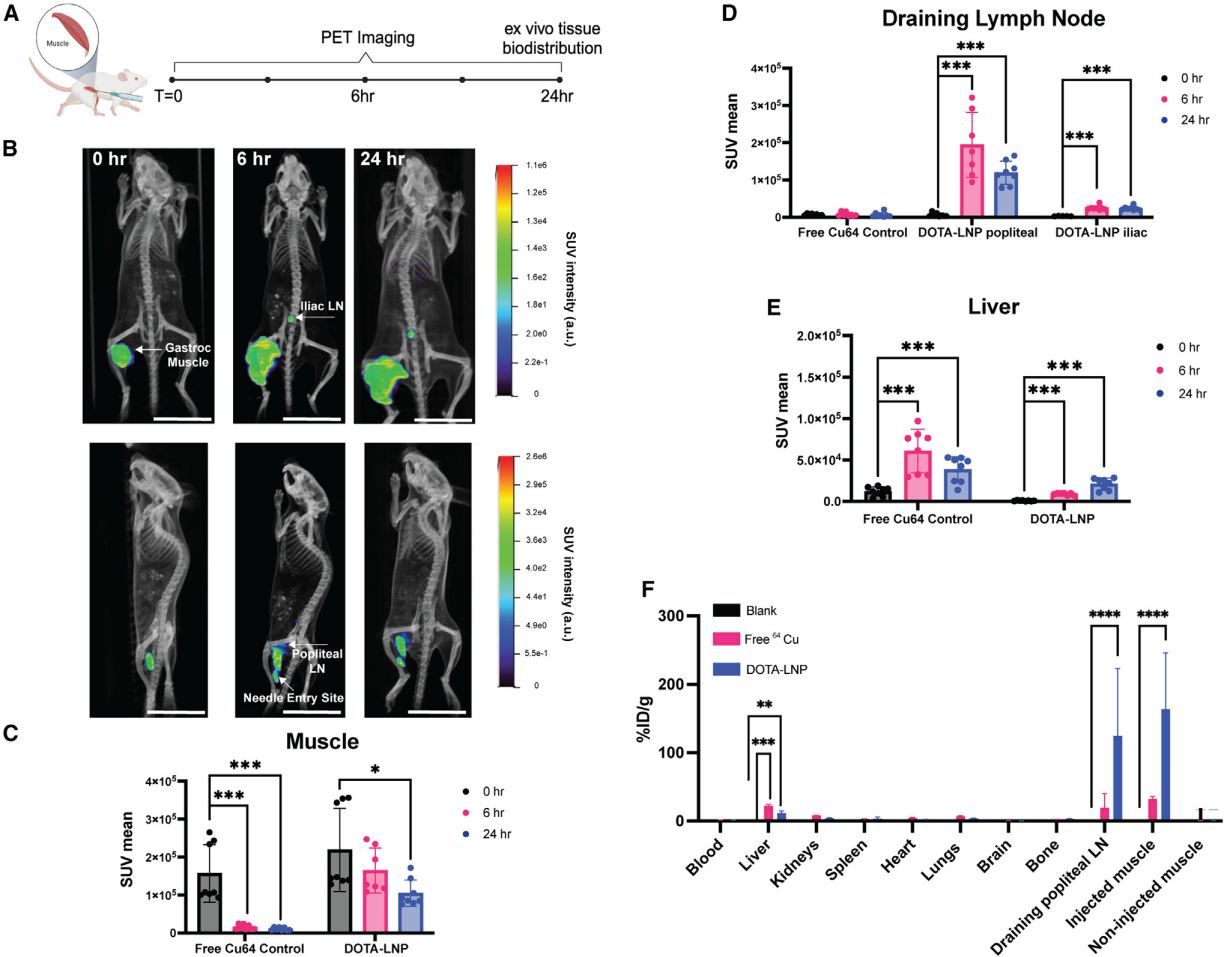
PET-CT imaging reveals LNPs primarily distribute at injected muscle and immediate draining lymph node in mice (A) PET-CT study timeline. DOTA-LNPs encapsulating 5 μg mCherry mRNA were administered i.m. into the gastrocnemius muscle of BALB/c mice (data pooled from two studies, one with *n* = 3 animals/group and one with *n* = 5 animals/group). (B) PET-CT projections of mice over the imaging time course. Two different thresholds/viewing angles are shown; upper panels show a higher sensitivity view to visualize lower LNP signals detected in iliac LNs, and lower panels show a lower sensitivity view to visualize the injection site and popliteal draining LNs. Scale bars, 50 mm. (C–E) ROI analyses of PET signal at injection site (gastrocnemius muscle, C), draining popliteal LNs (D), and liver (E) for animals receiving free ^64^Cu or ^64^Cu-loaded DOTA-LNPs. (F) E*x vivo* tissue gamma counter measurements of ^64^Cu signal from DOTA-LNPs compared with blank and free ^64^Cu controls. Statistical significance was determined by multiple unpaired t tests followed by Holm-Sidak post hoc test. ns, *p* > 0.05; ∗*p* < 0.033; ∗∗*p* < 0.002; ∗∗∗*p* < 0.001; ∗∗∗∗*p* < 0.0001. All data show means ± SEM.

We next employed PET imaging to track LNP biodistribution in live mice over time. To evaluate radiometal loading, ^64^Cu was added to DOTA-LNPs for 60 min followed by dialysis to remove unbound copper. Thin-layer chromatography analysis showed effective loading of the LNPs with ^64^Cu and removal of free metal (Figure S1D–S1F). To validate the utility of ^64^Cu/DOTA-labeling for assessing the biodistribution of LNP-mRNA, cohorts of three BALB/c mice were injected with 5 μg mCherry mRNA encapsulated in ^64^Cu-loaded DOTA-LNPs (Figure S1G) i.m. in the right gastrocnemius muscle. As a control, a separate cohort of mice was injected with free ^64^Cu (same activity and volume as DOTA-LNPs) to distinguish the behavior of free ^64^Cu. Animals were imaged via whole-body PET-computed tomography (CT) at 0 h, 6 h, and 24 h post-immunization; at 24 h, tissues were isolated for *ex vivo* PET imaging (Figure 2A). This study was then repeated in a second larger cohort of five animals and we present the analysis of data pooled from these two studies. Labeled LNPs were clearly visible dispersing in the injected muscle immediately following injection (Figures 2B and S3). Further, by 6 h, signals could be detected in multiple draining lymph nodes, and for some animals, LNPs were detected at the needle entry point in the muscle (suggesting some leakage along the needle track, Figure 2B). Popliteal LNs showed LNP signal in all of the mice, and iliac LNs showed LNP signal in most animals (seven out of eight mice) (Figures 2B and S3, Videos S2 and S3). Free copper by contrast showed no accumulation in draining nodes (Figure S3, Video S1). We quantified mean standard uptake values (SUVmean; PET signal normalized to dose and body weight) at the injection sites, draining popliteal LNs, and liver over time. Free copper cleared from the injection site almost entirely within 6 h, while DOTA-LNPs showed a slower decay, with ∼40% of the LNP signal initially deposited still present at 24 h (Figure 2C). Free copper showed no accumulation in draining LNs, while DOTA-LNP signal accumulated by 6 h in all animals and was still present at 24 h (Figure 2D). Such a rapid transport to LNs suggests direct drainage of LNPs via lymphatics, as it is too rapid to reflect cell-mediated transport. Signal from DOTA-LNPs in the liver was also seen to increase with time, but was an order of magnitude lower than signal detected in draining LNs and substantially lower than signal from the free ^64^Cu control (Figure 2E).

Video S1. Mouse PET-CT video projection for free 64Cu control (from left to right) at 0, 3, and 24 h timepoints

Video S2. Mouse PET-CT video projection at 0-h time point

The DOTA-LNP-injected animals were euthanized at 24 h and organs were harvested for *ex vivo* activity quantification via gamma counter. The only significant differences in signal between the blank control and DOTA-LNPs were seen in injected muscle and popliteal draining lymph node tissues, with minor liver signal remaining, which was lower than that of free ^64^Cu (Figure 2F). Last, we compared the signal clearance kinetics of LNPs labeled with the fluorescence tracer DiD vs. the clearance of PET signal from injected muscles, and saw very similar kinetics of signal decay, suggesting that loss of radioactive copper from the LNPs over the imaging time course is limited (Figure S4). Overall, PET-CT and gamma quantification analysis showed a strong trafficking preference for LNPs post-i.m. injection to distribute in the injected muscle and proximal draining lymph nodes, with relatively high retention of LNPs exhibited at the injection site, which was distinct from the behavior of free copper.

### LNPs distribute between the injection site and draining lymph nodes in rhesus macaques

**Figure 3:**
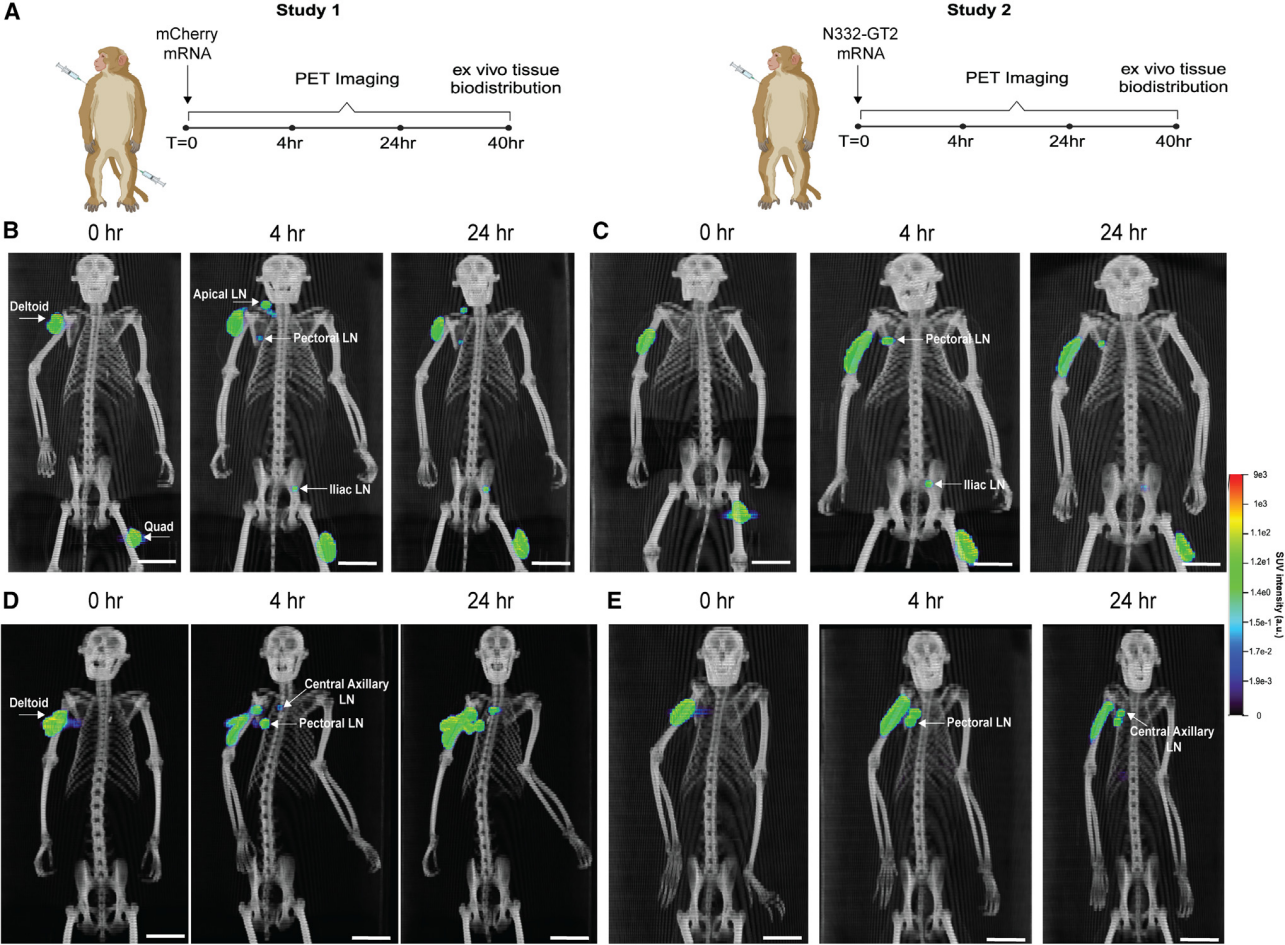
LNPs rapidly reach draining lymph nodes following i.m. administration in rhesus macaques (A) Schematics of PET-CT timelines for mCherry (left) and N332-GT2 (right) NHP studies. Animals received injections of 50 μg mRNA per site i.m. (B) PET-CT projections of one representative animal from the mCherry study over time. (C) PET-CT projections of a second representative animal from the mCherry study over time. (D) PET-CT projections of one representative animal from the N332-GT2 study over time. (E) PET-CT projections of a second representative animal from the mCherry study over time. Scale bars in B–E represent 50 mm.

We next evaluated the biodistribution of mRNA-loaded LNPs in rhesus macaques. DOTA-LNPs were loaded with radioactive ^64^Cu as before and mixed 1:1 with LNPs labeled with the lipophilic fluorophore DiD to enable whole-animal PET imaging followed by tissue-level biodistribution/imaging analyses. Both LNPs encapsulated mRNA either encoding for mCherry fluorescent protein or a transmembrane form of the stabilized HIV Env trimer N332-GT2.^7^ In a first set of four animals, LNPs carrying mCherry mRNA were injected in the left deltoid and right quadriceps (50 μg mRNA per injection site), with animals imaged via whole-body PET-CT at 0 h, 4 h, and 24 h (Figure 3A, left schematic, Videos S4, S5, and S6). In a second cohort of four animals, LNPs encapsulating N332-GT2 mRNA were administered only in the left deltoid, and followed the same PET-CT imaging timeline (Figure 3A, right schematic, Videos S7, S8, and S9).

Video S4. NHP mCherry study PET-CT video projection at 0-h time point

Video S5. NHP mCherry study PET-CT video projection at 4-h time point

Video S6. NHP mCherry study PET-CT video projection at 24-h time point

Video S7. NHP N332-GT2 study PET-CT video projection at 0-h time point

Video S8. NHP N332-GT2 study PET-CT video projection at 4-h time point

Video S9. NHP N332-GT2 study PET-CT video projection at 24-h time point

The whole-animal images revealed distinct draining LN basins for the deltoid vs. quadriceps injections. For the quadriceps injection site, at 4 h, LNP signal was distributed through the injected muscle, and could also be clearly detected in iliac LNs (Figures 3B and 3C; Figure S5, and Video S5). LNPs injected i.m. in the deltoid by contrast drained stochastically to axillary, apical, or pectoral lymph nodes, with different LNs exhibiting LNP uptake in different animals, (Figures 3B–3E; Figure S6, and Videos S5, S8). Although multiple lymph nodes may reside at each of these drainage sites, the resolution of the PET scans did not permit clear identification of whether one or more nodes took up vaccine at any of the individual drainage sites. Low signal was also detected in the spleen of two NHPs but was negligible in the majority of animals (Figures S5 and S6). LNP signals were not detected in the heart or other tissues.

**Figure 4:**
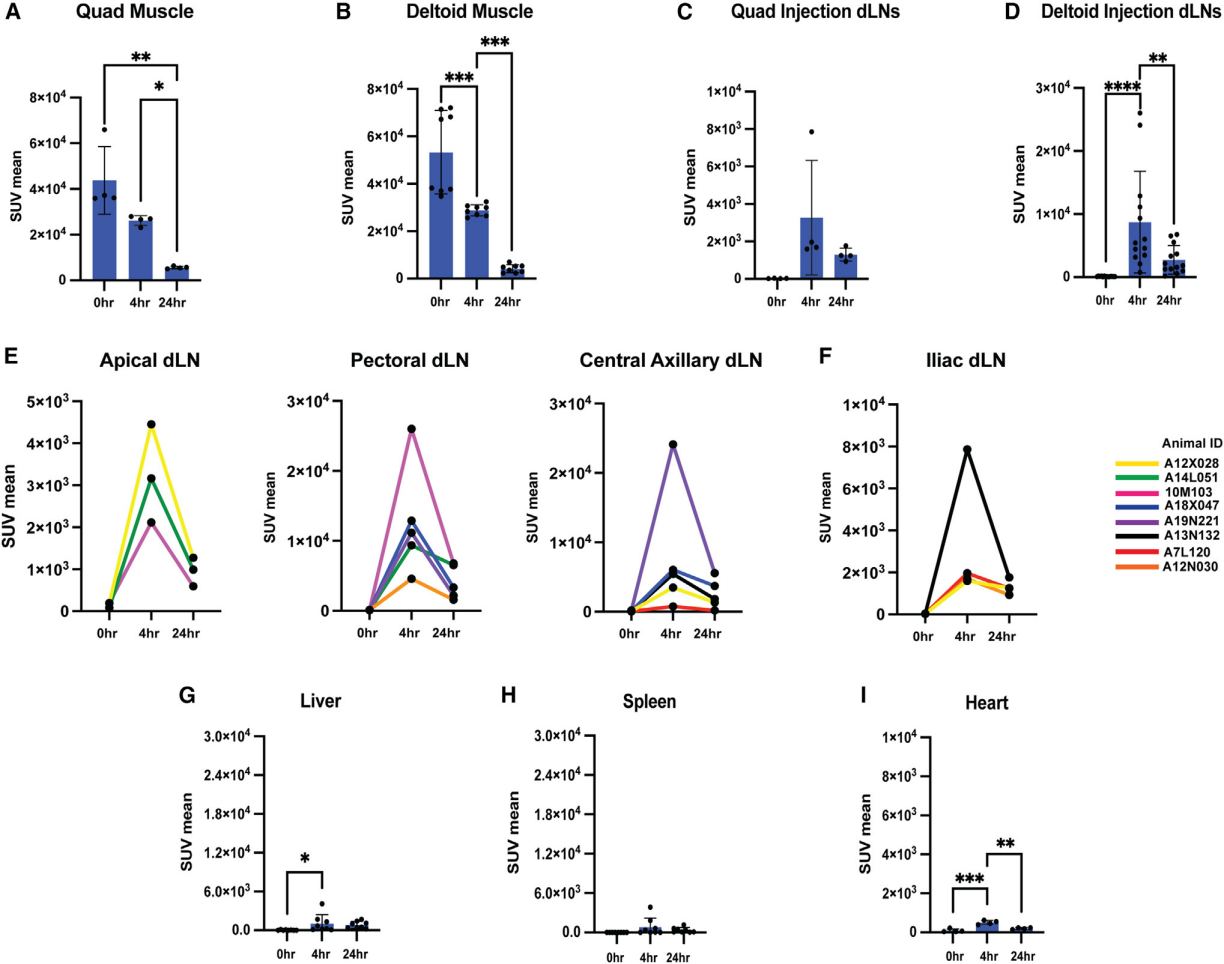
LNPs access draining lymph nodes but show very limited systemic distribution following i.m. injection in rhesus macaques (A–D) ROI analyses of PET signal at quadriceps muscle injection site (A), deltoid muscle injection site (B), quadriceps-draining lymph nodes (C), and deltoid-draining lymph nodes (D). (E and F) ROI analyses of PET signal in the deltoid-draining lymph nodes (apical, pectoral, and central axillary) (E) and the quadriceps-draining lymph node (iliac) (F) with different line colors corresponding to individual animals. (G–I) ROI analyses of PET signal at liver (G), spleen (H), and heart (I). Statistical significance was determined by one-way ANOVA followed by Tukey’s post hoc test. ns, *p* > 0.05; ∗*p* < 0.05; ∗∗*p* < 0.01; ∗∗∗*p* < 0.001; ∗∗∗∗*p* < 0.0001. All data show means ± SEM.

As the deltoid LNP biodistributions were similar in the two cohorts, we pooled the quantitative analyses of the muscle and dLNs for this injection site. Decay-corrected PET signals in the injected muscle sites steadily decreased from 0 to 24 h, suggesting dissemination of LNPs from the injection site (Figures 4A and 4B). Draining lymph nodes by contrast showed peak signal at 4 h, followed by a drop in LNP signal by 24 h (Figures 4C and 4D). From the deltoid injections, LNP uptake was stochastically detected in axillary, apical, and pectoral LNs, and a majority of animals (five out of eight) showed uptake in two different dLN sites (Figure 4E). Annotation of LNP uptake animal by animal showed that iliac LNs were preferentially targeted following quadriceps injection, and we only detected signals in single lymph node sites (Figure 4F). Consistent with our findings in mice, LNP signal in the liver was detectable but ∼10-fold lower than that detected in draining lymph nodes; and the signal detected in the spleen and heart was also very low (Figures 4G–4I). Thus, LNPs distribute primarily in the injected muscle and nearby draining lymph nodes early after mRNA immunization.

### Tissue-level analysis confirms LNPs and vaccine mRNA are delivered to multiple draining lymph nodes in NHPs

**Figure 5:**
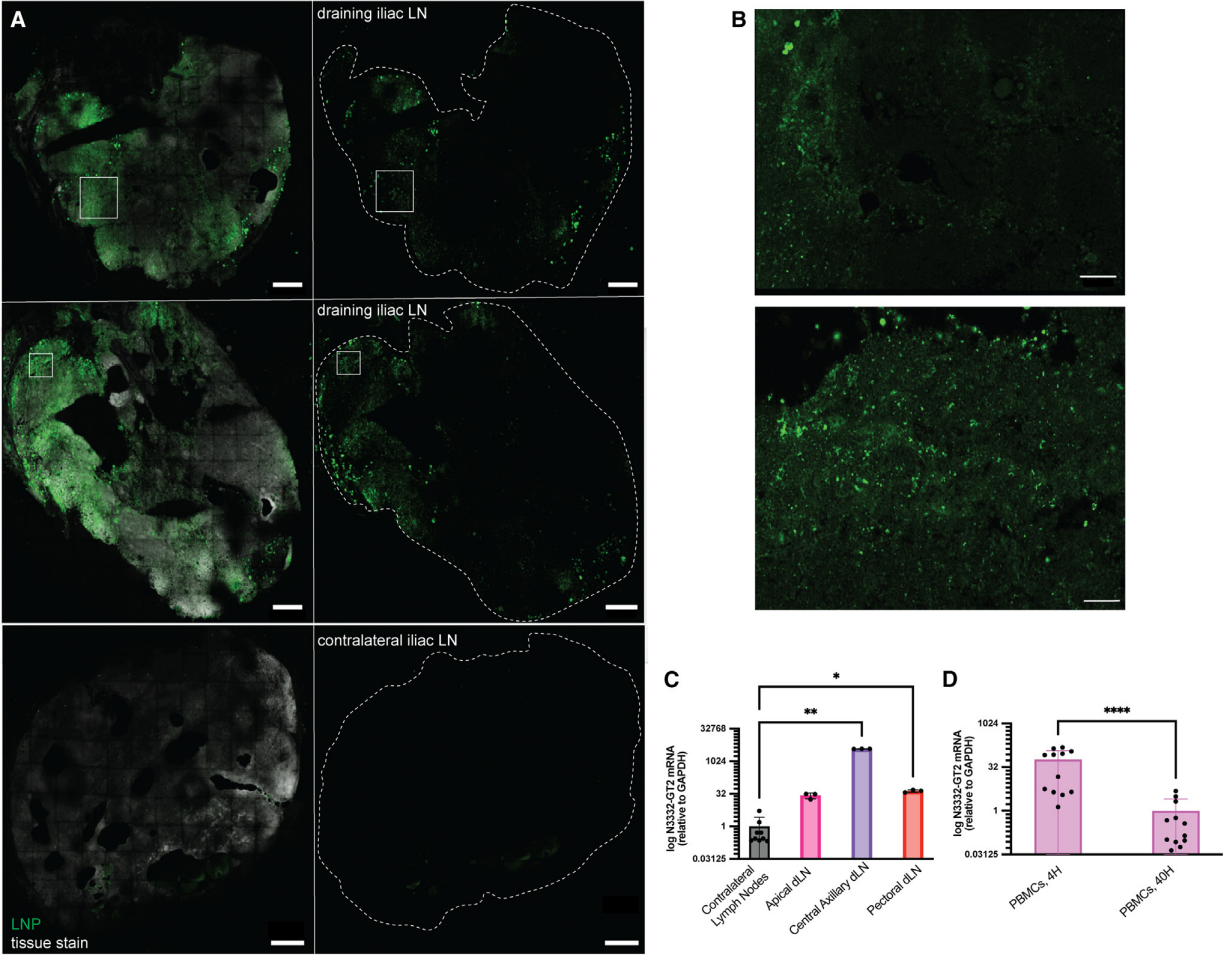
*Ex vivo* tissue analysis reveals LNPs and mRNA are persistent in draining lymph nodes of NHPs (A) NHP draining and non-draining (contralateral) lymph node samples taken at 40 h post-immunization. LNP signal via diD in *green*, tissue stain in *gray*. Imaging was conducted on 28 lymph node tissue samples collected from all quadriceps and deltoid-draining lymph node sites; shown are representative lymph node images. Magnification at 25× with left panel showing merged signal and right panel showing only LNP signal. Scale bars, 400 μm. (B) 63× magnification comparing LNP signal (green) between selected draining (white box indicating zoomed in region of view in 25× image) and non-draining lymph node samples. Scale bars 115 μm. (C) Expression of N332-GT2 mRNA in lymph nodes from ipsilateral (right) and contralateral (left) side measured by qRT-PCR using GAPDH as a reference gene. The experiment represents apical, central axillary, and pectoral lymph nodes obtained from a single animal 24 h post-injection. Three technical replicates were performed for each sample. (D) Expression of N332-GT2 mRNA in sorted PBMCs at 4 h and 24 h post-injection measured by qRT-PCR using GAPDH as a reference gene. These experiments represent PBMCs obtained from four animals. Three technical replicates were performed for each sample. Statistical significance was determined by Kruskal-Wallis test. ns, *p* > 0.05; ∗*p* < 0.0332; ∗∗*p* < 0.0021; ∗∗∗*p* < 0.0002; ∗∗∗∗*p* < 0.0001. All data show means ± SEM.

At 40 h post-injection, the four animals receiving LNPs carrying mCherry mRNA in the quadriceps were euthanized and draining iliac lymph nodes were harvested for tissue-level analysis. We first carried out confocal imaging of histological tissue slices to visualize the biodistribution of LNPs in quadriceps-draining iliac LNs. When compared with control contralateral lymph nodes, substantial LNP signal (green) was detected throughout the tissue sections (Figures 5A and 5B).

To confirm that mRNA was also delivered to dLNs, we carried out qPCR analysis to detect Env trimer mRNA in deltoid-draining LNs collected from the second PET study. Trimer mRNA was detected in axillary and pectoral lymph nodes, with the greatest amount detected in the central axillary lymph nodes (Figure 5C). In addition, qPCR was run on collected PBMCs (peripheral blood mononuclear cells) to assess mRNA in circulating cells at 4 h and 40 h post-injection. Trimer mRNA was detected in PBMCs from three of four animals at 4 h post-immunization but was undetectable at 40 h (Figure 5D). These findings corroborate the PET-CT imaging data suggesting LNP/mRNA reaches multiple draining lymph nodes at early time points post-immunization.

### APCs found to be cell type responsible for LNP uptake and mRNA translation in draining LNs

Following PET-CT imaging of the four animals receiving LNPs carrying mCherry mRNA, draining and contralateral control lymph node tissues were harvested at 40 h to investigate the cellular distribution of DOTA-LNPs and mRNA-encoded protein using flow cytometry (Figures S7 and S8). Fluorescent LNPs were readily detected in a small proportion of lymph node cells collected from draining lymph nodes at both the deltoid and quadriceps injection sites (Figures S9A and S9B). Consistent with the PET-CT findings indicating a greater frequency of animals exhibiting LNP trafficking to iliac LNs, LNPs injected at the quadriceps were primarily detected in cells from iliac but not nearby inguinal nodes (Figures S9A and S9B). From LNs analyzed at the deltoid injection site, LNP uptake was primarily detected in apical and pectoral LNs; however, only two of the deltoid-draining LNs we analyzed showed a relatively high (>1%) LNP signal, limiting our ability to draw significant conclusions for drainage at this area (Figures S9A and S9B). Although we expected mRNA expression to be substantially decayed by the time point these tissues were collected (40 h post-immunization), we also assessed mCherry signal in LN cells. Low but statistically significant mCherry expression was detected in cells from the quadriceps-draining iliac LNs, and in two apical LNs draining the deltoid immunization site (Figures S9C and S9D).

To examine which cell populations were taking up LNPs, we first assessed changes in myeloid cell populations in the dLNs that may have been induced as part of the innate immune response triggered by LNP-mRNA immunization.^16^^,^^17^^,^^18^ Interestingly, in the quadriceps-draining LNs, myeloid DCs (mDCs) and monocytes were significantly increased relative to the contralateral control nodes (Figure S10A). There was a trend toward increased mDCs and monocytes detected in deltoid-draining LNs relative to contralateral control LNs, but these changes did not reach statistical significance (Figure S10B). We next examined the association of LNP and mCherry signal with different LN cell populations. In the quadriceps-draining LNs, myeloid DCs and monocytes showed the highest uptake of LNPs, and these were the cell types that also were detected with mCherry expression, along with a small population of neutrophils (Figures S9E and S9F). LNP uptake was also detected in CD4^+^ T cells and B cells, but mCherry^+^ cell numbers were too low to draw any conclusions about expression in these cell types (Figure S9G and data not shown). An analogous analysis of the deltoid-draining LNs showed similar trends for LNP uptake in mDCs and monocytes, but these observations did not reach statistical significance (Figures S10C–S10F). Notably, we found that APCs that were LNP^+^ also upregulated the activation marker CD80 (Figure S9H) and to a lesser extent CD40 and MHCII (Figures S10G and S10H) relative to LNP^−^ cells within the same LN. Altogether, these data indicate that a variety of cells with antigen-presenting/phagocytic functions are the primary cells acquiring LNPs.

## Discussion

mRNA delivered by lipid nanoparticles has emerged as an important clinical modality for vaccines, with millions of doses safely administered during the COVID pandemic. However, much of the underlying mechanisms of action for mRNA vaccines *in vivo* remain to be understood. Here we sought to gain insights into the early biodistribution and kinetics of LNP distribution in tissues in rhesus macaques, an important preclinical animal model for vaccine development and the closest animal model genetically to humans. Using whole-animal PET-CT imaging and testing two administration sites, we identified several features of LNP pharmacokinetics following intramuscular injection: First, LNPs spread within the injected muscle tissue immediately following injection, but also rapidly showed uptake in distinct draining lymph node pools. Second, the distribution of LNPs among different draining lymph node basins was stochastic animal to animal, particularly for deltoid i.m. injections, where three different lymph node regions—axillary, apical, and pectoral LNs—were variably involved animal to animal. Third, lymph node accumulation was observed within 4 h post-injection, a time span suggesting that direct transport through lymphatics plays a role in LNP accumulation in dLNs. This accumulation of LNPs in lymph nodes observed by live animal imaging was corroborated by histological and flow cytometry analyses at later time points.

In preliminary studies in mice, we found, similar to our data in NHPs, LNPs rapidly distributed both in the injected muscle and proximal draining lymph nodes, with negligible signal in other tissues except for low levels of uptake in the liver. Previous studies investigating LNP biodistribution in mice and rats have identified the liver as a site for LNP accumulation at early time points post-injection, following both intravenous and i.m. injections.^19^^,^^20^ We also detected LNPs accumulating in the liver in mice, albeit at lower levels than the draining lymph node and lower than the accumulation occurring upon free copper injection.

In macaques, we found that LNPs are rapidly detectable in certain draining lymph node basins. In agreement with this finding, a study of lipoplexes formed by complexation of mRNA with an aminoglycoside lipid CholK administered in the quadriceps of macaques and imaged by PET-CT whole-animal imaging detected RNA trafficking to multiple lymph nodes distal to the injection site within 4 h.^21^ The lipoplexes in this PET study have very different particle size, charge, and surface chemistry than the LNPs approved for use in humans, and thus it remains unclear if the rapid transport to LNs observed with this study is relevant for human mRNA/LNP vaccines. Such early signal detection in the draining lymph nodes is too rapid to reflect cell-mediated transport and is most consistent with direct transport of LNPs via lymph. Cell-mediated trafficking of LNPs/mRNA from the injection site could still occur over a period of multiple days post-immunization, which is beyond the time frame that our ^64^Cu radiotracer could reliably report. Although we saw decays in LN LNP signal between 4 and 24 h, fluorescently tagged LNPs were still readily detected by histology in these tissues 40 h post-injection.

The stochasticity in terms of which dLNs showed LNP uptake from the deltoid injection site appears to be a feature of the NHP model that may be distinct from humans, as longitudinal fine needle aspirate studies of volunteers receiving the COVID-19 mRNA vaccines have been quite successful in detecting robust ongoing germinal center responses simply by sampling draining axillary LNs.^22^^,^^23^ In humans, vaccine mRNA has been detected in biopsied axillary lymph nodes and blood of healthy volunteers, and at low levels in heart tissue but not liver or spleen of recently deceased vaccinees by RNA FISH (fluorescence *in situ* hybridization) and qPCR.^24^^,^^25^ At the protein level, vaccine antigens have been transiently detected at low levels in the blood of human vaccinees, and also localized in germinal centers of dLNs following booster immunization with the mRNA COVID vaccines.^26^ We detected little or no LNP signal in heart tissue via PET imaging following i.m. immunization of NHPs. In alignment with what we observed in NHPs by PET, no vaccine mRNA was detected in the liver in humans.^24^

A limitation of these studies is that to enable DOTA tagging, we employed LNP formulations mimicking the Moderna COVID vaccine LNP, but these were by necessity fabricated by labscale microfluidic mixing and do not necessarily exactly match the properties of current clinical LNPs. However, DLS analysis of the approved Pfizer and Moderna mRNA LNP COVID vaccines have reported that these clinical formulations have similar polydispersity indices to the LNPs used here (∼0.2), and while the Z-average particle size of the Pfizer LNPs are reported to be somewhat less than 100 nm, the Moderna LNPs have been reported to have a Z-average slightly above 200 nm.^27^^,^^28^ Hence, we expect the LNPs studied here are a reasonable surrogate for LNPs in clinical use. Another limitation in this imaging study is the short half-life of ^64^Cu (12.7 h^29^), and hence we restricted our live animal imaging time course to 24 h, when the PET signals were still strong. As free copper rapidly accumulates in the liver (Figure 2E), but very low PET signals were detected in the liver in our NHP imaging studies, we infer that disassociation of copper from the LNPs is not substantial over the imaging time course. LNP biodistribution patterns seen by whole-animal PET imaging were corroborated by flow cytometry and histological imaging analysis of individual draining lymph nodes. mRNA vaccines have been reported to trigger an increase in myeloid cells in draining lymph nodes that are capable of taking up LNPs and initiating an innate immune response.^12^ In accordance with these observations, we found myeloid cells to increase in mRNA-vaccinated animals, with APCs primarily responsible for LNP uptake. Additionally, monocytes, neutrophils, and DCs were similarly identified as cells responsible for translating vaccine mRNA. These findings are largely in agreement with prior work that characterized LNP uptake and mRNA expression by flow cytometry in NHP lymph nodes at earlier time points (24 h and earlier).^12^^,^^13^

In summary, PET-CT imaging is a powerful modality to gain a whole-animal level view on the biodistribution of vaccines in NHPs. Using metal chelator-conjugated lipids, we were able to visualize LNP/mRNA vaccine biodistribution in both mice and NHPs. These studies identified stochastic transport to proximal draining lymph nodes with limited distribution into other tissues. These data provide further insights into the key lymph nodes involved in the immune response to mRNA vaccines in the closest animal model to humans, and can guide further mechanistic studies of key draining lymph nodes where mRNA vaccines act.

## Materials and methods

### mRNA synthesis

Template DNA plasmids used in the production of mRNA were created using a commercially available Cloning Kit for mRNA Templates (Takara #6143) according to the manufacturer’s instructions. Resultant plasmid DNA was linearized via endonuclease digestion and purified with PureLink PCR Purification columns (ThermoFisher #K310002) following the manufacturer’s instructions. To synthesize RNA, 20 μL *in vitro* transcription (IVT) reactions were performed using reagents from the HiScribe T7 High Yield RNA Synthesis Kit (NEB #E2040) and 1–2 μg of linear DNA template (scaled as needed). Modified base N1-methylpseudouridine triphosphate (TriLink #*N*-1081) was added to the reaction mixture instead of canonical uridine triphosphate, and CleanCap Reagent AG (TriLink #*N*-7113) was utilized to co-enzymatically add 5′ Cap-1 structures to synthesized RNA. The IVT product was purified using PureLink RNA Mini columns (ThermoFisher #12183018A) following the manufacturer’s instructions. Quality of the resulting mRNA was assessed using UV-Vis spectrophotometry and gel electrophoresis.

### DSPC-DOTA synthesis

1,2-distearoyl-sn-glycero-3-phosphocholine (N-azidoethyl) (18:0 azidoethyl PC, Avanti Polar Lipids) was dissolved in chloroform at 10 mg/mL and reacted with a 3-fold molar excess of methanol-dissolved BCN-DOTA (CheMatech). The reaction was allowed to occur for at least 1 day at 4°C with shaking at 5 mg/mL lipid in a 50:50 mixture of chloroform and methanol. After completion of the reaction, DSPC-DOTA was purified from excess BCN-DOTA via reverse-phase high-pressure liquid chromatography (RP-HPLC) on a Jupiter C4 column (5-μm particles, 300 Å – Phenomenex P/N: 00G-4167-E0) following the gradient shown in Table S1. Fractions corresponding to DSPC-DOTA were collected and methanol was removed on a rotovap. The sample was then diluted with a 10× volume of deionized water, frozen in liquid nitrogen and lyophilized.

### mRNA encapsulation in LNPs

Lipids were stored in ethanol at −20°C, and RNA constructs were stored in RNAse-free water at −80°C and were thawed on ice before use. The two phases were prepared at an ethanol:aqueous volume ratio of 1:2, and RNA and lipids combined at an N:P ratio of 5:1. Each phase was loaded into a syringe (BD) and locked onto the NxGen microfluidic cartridge for mixing using a NanoAssemblr Ignite instrument (Precision Nanosystems). The Ignite was set to operate with the following settings: volume ratio- 2:1; flow rate- 12 mL/min; waste volume- 0 mL. The resulting LNPs were then dialyzed into pH 7.4 PBS using Slide-A-Lyzer MINI dialysis devices with a 20K molecular weight cutoff for two rounds at 45 min per round.

The organic phase was prepared by solubilizing the lipids SM102 (BroadPharm CAT#25499), DSPC (Avanti Polar Lipids CAT#850365), cholesterol (Avanti Polar Lipids CAT#700100), DMG-PEG2k (Avanti Polar Lipids CAT#88015), and DSPC-DOTA (see DSPC-DOTA synthesis) in ethanol at a molar ratio of SM102:DSPC:cholesterol:DMG-PEG2k:DOTA 50:9.5:38.5:1.5:0.5 and a total lipid concentration of 5 mg/mL. For non-DOTA lipids, SM102, DSPC, cholesterol, and DMG-PEG2k were solubilized in ethanol at a molar ratio of 50:10:38.5:1.5 and a total lipid concentration of 5 mg/mL. Non-DOTA lipids labeled with fluorescent diD were solubilized in ethanol at a molar ratio of SM102:DiD:DSPC:cholesterol:DMG-PEG2k 50:0.1:9.9:38.5:1.5 and a total lipid concentration of 5 mg/mL. The aqueous phase of RNA was prepared by diluting the RNA (stored in RNAse-free water) with 10 mM citrate buffer at pH 3.0 (CAT#J61391-AK; Alfa Aesar) such that the mixture had an RNA concentration of 0.10 mg/mL.

### LNP characterization

Size distributions of LNPs (2 μg/μL mRNA final concentration) in deionized water were analyzed by dynamic light scattering (DLS) using a Brookhaven Malvern Panalytical DLS system.

For cryoTEM imaging, LNPs were dialyzed in RNAse-free water using Slide-A-Lyzer MINI dialysis devices with a 20K molecular weight cutoff for two rounds at 1 h per round. After dialysis, LNPs were diluted in RNAse water to a concentration of 2 μg/mL. In sample preparation for cryogenic electron microscopy (cryo-EM), 3 μL of the particle sample in buffer containing solution was dropped on a lacey copper grid coated with a continuous carbon film and blotted to remove excess sample without damaging the carbon layer by Gatan Cryo Plunge III. The grid was then mounted on a Gatan 626 single tilt cryo-holder equipped in the TEM column. The specimen and holder tip were cooled down by liquid nitrogen, and the temperature was maintained during transfer into the microscope and subsequent imaging. Imaging on a JEOL 2100 FEG microscope was conducted using a minimum dose method that is essential to avoid sample damage under the electron beam. The microscope was operated at 200 kV and with a magnification in the ranges of 10,000–60,000 for assessing particle size and distribution. All images were recorded on a Gatan 2kx2k UltraScan CCD camera.

### C2C12 cells *in vitro* transfection

C2C12 murine myoblast cells (ATCC) were cultured in Dulbecco’s Modified Eagle’s Medium (DMEM) containing 10% fetal bovine serum (FBS) and seeded in a six-well plate at a density of 1 × 10^6^ cells per well. On the day of transfection, cells were washed two to three times in PBS and incubated with 50 μL of mCherry mRNA (concentration 0.1 mg/mL) encapsulated in LNPs or DOTA-LNPs diluted in optim-MEM at 37°C for 4–6 h. After 4–6 h, cell media was added on top of treatment and cells were placed back in 37°C for 24 h. At 24 h post-transfection, cells were plated in a 96-well U-bottom plate, stained with AquaZombie live/dead stain for 15 min at 25°C, and resuspended in flow cytometry buffer then analyzed on a BD FACSCelesta Cell Analyzer.

### Animals and ^64^Cu-labeled LNP immunizations

All animal studies were carried out following an Institutional Animal Care and Use Committee (IACUC)-approved protocol following state, local, and federal guidelines. DOTA-LNPs were incubated with ^64^Cu (CuCl_2_ in 0.1N NaOHI, 30 μCi activity per 10 μg mRNA-encapsulated DOTA-LNPs) buffered in pH 7.4 PBS obtained from the Mallinckrodt Institute of Radiology at Washington University School of Medicine at 25°C in a designated radioactivity space. Following 1-h incubation, loaded LNPs were dialyzed into PBS using Slide-A-Lyzer MINI dialysis devices with a 20K molecular weight cutoff for two rounds at 45 min per round. After dialysis was completed, LNPs were measured for radioactivity (readout in mCi) and loading was validated by instant thin-layer chromatography analysis. Eight-week-old BALB/c mice (Jackson Laboratories) were injected intramuscularly in the right gastrocnemius with 10 μg mRNA encapsulated in ^64^Cu -loaded DOTA-LNPs administered in 50 μL PBS. The total radioactivity injected was 12.7–13.7 μCi. Radioactive animals were housed in a separate, designated room, according to Environmental Health & Safety policies, until 10 half-lives had elapsed (5.3 d for ^64^Cu).

Indian rhesus macaques were maintained in accordance with the regulations of the Guide for the Care and Use of Laboratory Animal at New Iberia Research Center, University of Louisiana at Lafayette. DOTA-LNPs were incubated with ∼4 mCi ^64^Cu (CuCl_2_ in 0.1N NaOHI) buffered in pH 7.4 PBS obtained from the Department of Medical Physics at the University of Wisconsin in Madison at 37°C in a designated radioactivity space. Following 1-h incubation, loaded LNPs were dialyzed into PBS using Slide-A-Lyzer MINI dialysis devices with a 20K molecular weight cutoff for two rounds at 45 min per round. After dialysis was completed, LNPs were measured for radioactivity (readout in mCi). In the first cohort of four animals, each animal was injected intramuscularly in the left quadriceps and right deltoid with 25 μg mCherry-encoding mRNA encapsulated in DOTA-LNP and 25 μg mCherry-encoding mRNA encapsulated in diD-labeled LNP in 500 μL PBS. The total radioactivity injected was 3.0 mCi. In the second cohort of four animals, each animal was injected intramuscularly in the right deltoid with 25 μg N332-GT2 trimer-encoding mRNA encapsulated in DOTA-LNP and 25 μg N332-GT2 trimer-encoding mRNA encapsulated in diD-labeled LNP in 500 μL PBS. The total radioactivity injected was 3.0 mCi. Radioactive animals were housed in a separate, designated room, according to EH&S policies, with daily radioactive measures, until 10 half-lives had elapsed (5.3 d for ^64^Cu).

### PET-CT imaging of mice and *ex vivo* tissue biodistribution

Immunized mice were imaged at 0, 3, 6, and 24 h post-injection using a G8 PET-CT preclinical small-animal scanner (PerkinElmer). Mice were anesthetized with isoflurane (2% mixed with oxygen) and kept warm using controlled heating pads during the PET-CT scan. Animals were imaged with a static PET scan for 10 min followed by a 1.5-min μCT for anatomical reference. Images were reconstructed with the default 3D maximum likelihood estimation method with CT attenuation correction. At 24 h, animals were euthanized and tissues were collected for *ex vivo* LNP biodistribution analysis using a Wizard2 automatic gamma counter (PerkinElmer). Measured activity standards were used to calibrate counts to injected dose measurements. Tissues were weighed individually and decay corrected as needed.

### PET-CT imaging of non-human primates

PET-CT scans were performed on a Philips Gemini TF16 scanner. Animals were imaged live at 0, 4, and 24 h post-injection, and following euthanasia at 40 h, select tissues were harvested for *ex vivo* imaging. Acquisitions were done in three-dimensional (3D) mode with an axial field of view of 57.6 cm.

### PET-CT imaging analysis

PET-CT data were analyzed using the Multi-Image Analysis GUI (MANGO, Research Imaging Institute, UT Health San Antonio) and AMIDE software. Decay-adjusted PET images (corrected to the time of vaccine injection) were normalized by the injected doses and body weight to generate standardized uptake value (SUV) maps. To designate regions of interest (ROIs), spherical outlines were placed on target tissues, freehand outline drawings were made, and/or tissue contours on the SUV maps were identified through signal thresholding. These ROIs were then subjected to statistical evaluations to determine SUV max, SUV mean, and SUV sum. The 3D image data are exhibited as color-coded maximum intensity projections (MIPs) of the SUV max maps.

### NHP tissue extraction, immunofluorescence tissue imaging

Tissue samples were flash frozen, embedded in OCT, and cryosectioned at a thickness of 50 μm. Sectioned slides were fixed for 10 min at 25°C with 4% paraformaldehyde, washed with PBS, and perimeters were drawn around samples with a delimiting hydrophobic pen. Samples were treated with Universal Fc receptor blocker (NB309-30) for 30 min at 4°C, followed by goat serum blocking buffer incubation for 60 min at 25°C. Goat anti-human immunoglobulin (Ig)D primary antibody (Southern Biotech Cat No. 2030-30) was diluted in blocking buffer and added to tissue samples at 0.02 mg/mL for 24 h at 4°C. After primary antibody incubation, samples were rinsed three times with PBS, and mounted with Prolong Diamond Antifade Mountant (Fisher Scientific Cat No. P36970). Slides were left to cure overnight at 4°C and stored at 4°C until imaged.

Tissue sections were imaged with a 25× water objective or 63× oil objective for immunofluorescence on a Leica SP8 Laser Scanning Confocal Microscope.

### Flow cytometry of lymph nodes

Lymph nodes of vaccinated macaques were isolated 40 h post-immunization, at the completion of PET imaging. The collected lymph nodes were first scanned before being mechanically dissociated into single-cell suspensions. Cells were filtered, counted, and resuspended in freezing medium before storage in liquid nitrogen for subsequent analysis by flow cytometry and single-cell RNA sequencing.

For flow cytometry analysis, Zombie UV fixable viability dye (BioLegend) was used according to manufacturer’s protocol. Cells were washed and incubated with Human TruStain FcX (Fc Receptor Blocking Solution) for 15 min at 25°C, followed by staining with a cocktail of fluorescent antibodies for 30 min at 4°C (panels 1 or 2 described below). After staining, cells were fixed with 4% paraformaldehyde for 20 min at 4°C. After staining, samples were spiked with Precision Count Beads and cell numbers were calculated according to the manufacturer’s protocol. Samples were acquired on an FACSymphony A3 (BD Biosciences) and data were analyzed using FlowJo v10 (FlowJo Inc).

Panel #1 included anti-human CD16 BUV396 (3G8, BD Biosciences, 1:20), CD14 BUV737 (M5E2, BD Biosciences, 1:20), CD123 BV421 (6H6, BioLegend, 1:20), CD80 BV650 (L307.4, BD Biosciences, 1:20), HLA-DR FITC (Tu36, BD Biosciences, 1:20), CD40 (5C3, BioLegend, 1:10), CD11c PE-Cy7 (3.9, BioLegend, 1:20), CD66abce APC-Vio770 (TET2, Miltenyi, 1:20).

Panel #2 included anti-human CD20 BUV737 (L27, BD Biosciences, 1:20), CD80 BV650 (L307.4, BD Biosciences, 1:20), PD1 BUV785 (EH12.2h7, BioLegend, 1:10), CD4 AF488 (OKT4, BioLegend,1:20), HLA-DR PE (Tu36, BioLegend, 1:20), CXCR5 PE-CY7 (Mu5UBEE, Invitrogen, 1:10), CD8 APC-Cy7 (RPA-T8, BioLegend, 1:50), CD14 APC-Cy7 (M5E2, BioLegend, 1:50), CD16 (3G8, BioLegend, 1:50). For both panels we also collected fluorescent signal for mCherry and the lipophilic dye DiD (APC channel).

### Statistics

All values are presented as means ± SEM. Statistical analysis was performed using either a two-way analysis of variance (ANOVA) with Tukey’s post hoc test (for NHP ROI analysis and *in vivo* flow cytometry) or a one-way ANOVA followed by Tukey’s post hoc test (for *in vitro* flow cytometry and mouse ROI analysis) (∗*p* < 0.05, ∗∗*p* < 0.01, ∗∗∗*p* < 0.001, and ∗∗∗∗*p* < 0.0001) or Mann-Whitney test (for qRT-PCR data) (ns, *p* > 0.05; ∗*p* < 0.0332; ∗∗*p* < 0.0021; ∗∗∗*p* < 0.0002; ∗∗∗∗*p* < 0.0001). All statistical tests and graphical figures were generated using GraphPad Prism Version 10.0.3.

## Data and code availability

All data supporting this study are included within the article and/or supporting materials.

## Acknowledgments

We thank the Koch Institute Swanson Biotechnology Center’s Flow Cytometry, Microscopy, Histology, PMIT, and Nanotechnology Materials core facilities for their technical support. This work was supported in part by the 10.13039/100016872Koch Institute Support (core) Grant 5P30-CA014051 from the 10.13039/100000054National Cancer Institute, the 10.13039/100000002NIH (award AI176533 to D.J.I. and award UM1AI144462 to W.R.S. and D.J.I.), and the Ragon Institute of MGH, 10.13039/100006919MIT, and Harvard. We extend our thanks to Douglas Ferrell for assistance in PET/CT scanning of non-human primates and the staff at the New Iberia Research Center for their expert care of the non-human primates.

## Author contributions

M.B. performed DOTA-LNP formation and mRNA encapsulation, LNP QC protocols, C2C12 cell transfection, *in vitro* flow cytometry, i.m. injections and necropsies for mouse study, PET-CT data analysis, *in vivo* flow cytometry processing and analysis, tissue staining, imaging, and analysis for IF, and wrote the manuscript. L.M. performed NHP shipping and tissue handling, *in vivo* flow cytometry, and qPCR processing and analysis, and edited the manuscript. I.S.P synthesized and validated DSPC-DOTA via HPLC and mass spectrometry. B.J.K. helped develop the DOTA-LNP formulation and optimized QC protocols for LNPs. K.K.M. performed qPCR RNA extraction and processing. J.D. produced the mRNA constructs used. K.Q. performed qPCR RNA extraction and processing. H.M. performed ^64^Cu loading, mouse PET-CT imaging and necropsies, and *ex vivo* tissue biodistribution processing and analysis. M.A. performed PET imaging and necropsy. F.V. designed the experiments and performed PET imaging, animal care, and necropsies. J.M.S. and W.R.S. provided the N332-GT2 gp151 immunogen amino acid sequence. D.J.I. designed the experiments and edited the manuscript.

## Declaration of interests

J.M.S. and W.R.S. are inventors on patent applications regarding N332-GT2 immunogens. W.R.S. is an employee of Moderna, Inc; however, the contributions from W.R.S. were made prior to his employment at Moderna.

## Supplemental information

Document S1. Figures S1–S10 and Table S1

Video S3. Mouse PET-CT video projection at 24-h time point

Document S2. Article plus supplemental information
