# Supplementary material for: Visualizing lipid nanoparticle trafficking for mRNA vaccine delivery in non-human primates
Source: Mol Ther. 2025 Jan 10;33(3):1105–17. doi: 10.1016/j.ymthe.2025.01.008 (PMC11897755; doi:10.1016/j.ymthe.2025.01.008)
Supplement: Document S1. Figures S1–S10 and Table S1 [file mmc1.pdf]

YMTHE, Volume *33*

Supplemental Information

Visualizing lipid nanoparticle trafficking

for mRNA vaccine delivery in non-human primates

Maureen Buckley, Mariluz Araínga, Laura Maiorino, Ivan S. Pires, B.J. Kim, Katarzyna Kaczmarek Michaels, Jonathan Dye, Kashif Qureshi, Yiming J. Zhang, Howard Mak, Jon M. Steichen, William R. Schief, Francois Villinger, and Darrell J. Irvine

Table S1. RP-HPLC parameters for purification of DSPC-DOTA.

**Column**	Jupiter C4	
**Column Temperature**	40 °C	
**Aqueous Phase (A)**	0.1 M TEAA Water	
**Organic Phase (B)**	Methanol	
**Flow Rate**	1 ml/min	
**Gradient**	Time	%B
	0	10
	4	10
	32	100
	45	100
	46	95
	54	95
	55	10
	60	10

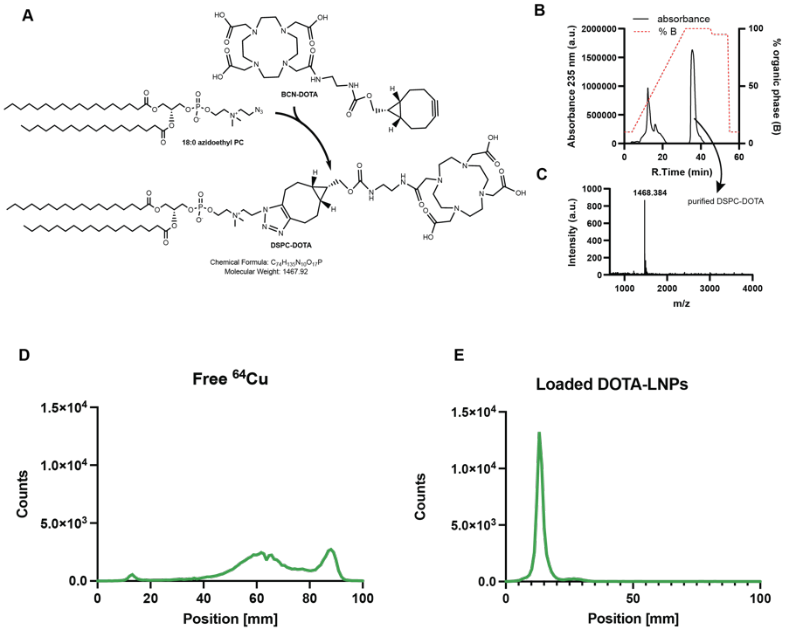

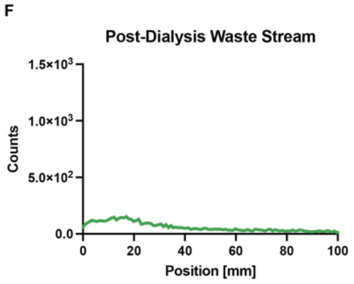

G

Group	Activity
DOTA-LNP	13.7 +/- 0.3
64Cu Con-	12.7 +/- 0.6

Figure S1. DSPC-DOTA synthesis, quality validation, and LNP ^{64}Cu loading validation. (A) Reaction scheme for DSPC-DOTA synthesis. **(B)** post-reaction purification via RP-HPLC **(C)** final purified product validation with mass spectrometry. **(D)** Thin layer chromatography analysis for free ^{64}Cu in PBS. **(E)** Thin layer chromatography analysis for waste steam post-LNP dialysis completed after ^{64}Cu loading shows minimal trace of free ^{64}Cu. **(F)** Thin layer chromatography analysis of loaded DOTA-LNPs traces one clean peak that marks successful loading. **(G)** Measured activity in microcuries for DOTA-LNP and free ^{64}Cu doses injected for PET-CT study

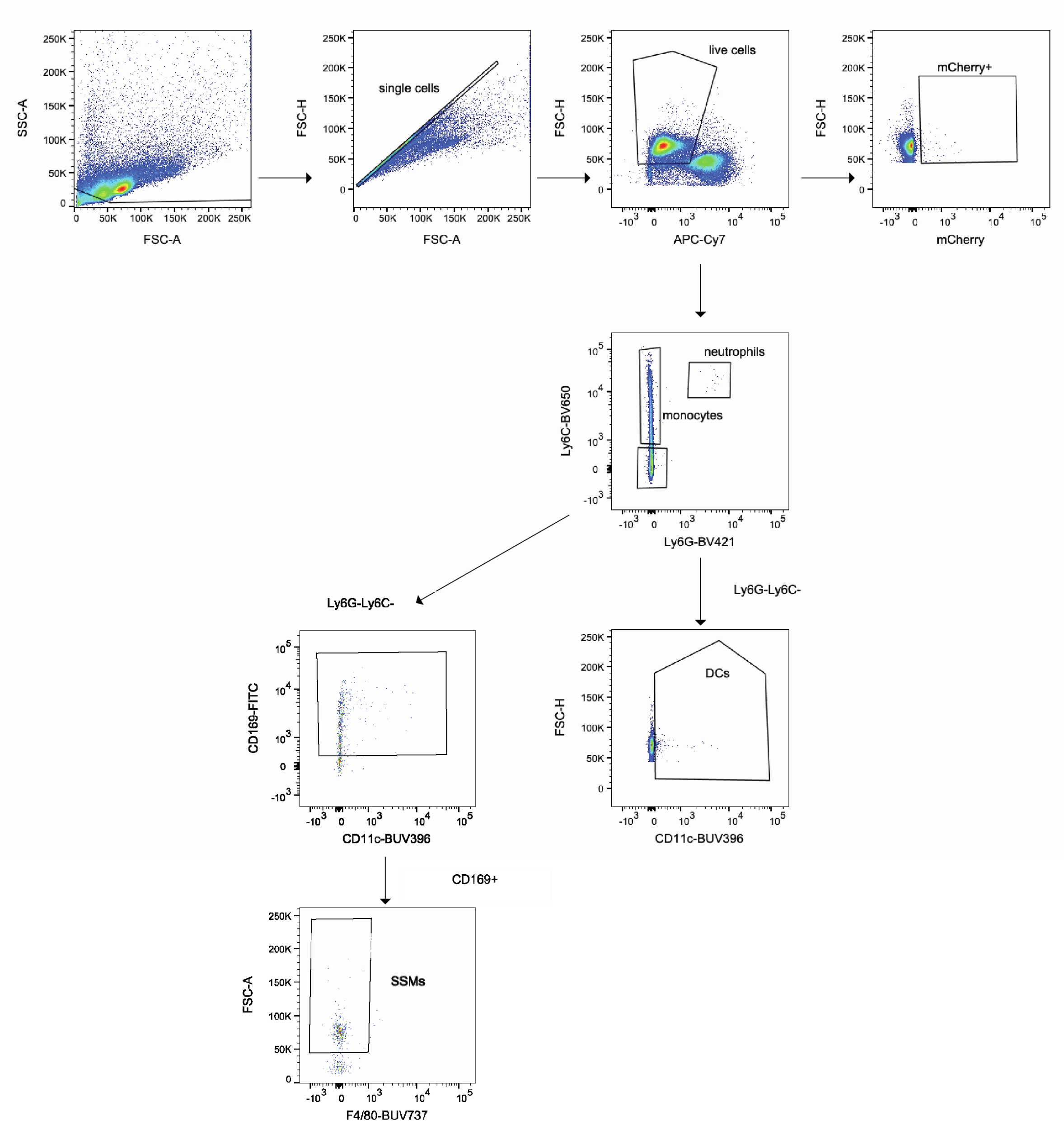

Figure S2. Flow cytometry gating strategy for mouse myeloid cell analysis.

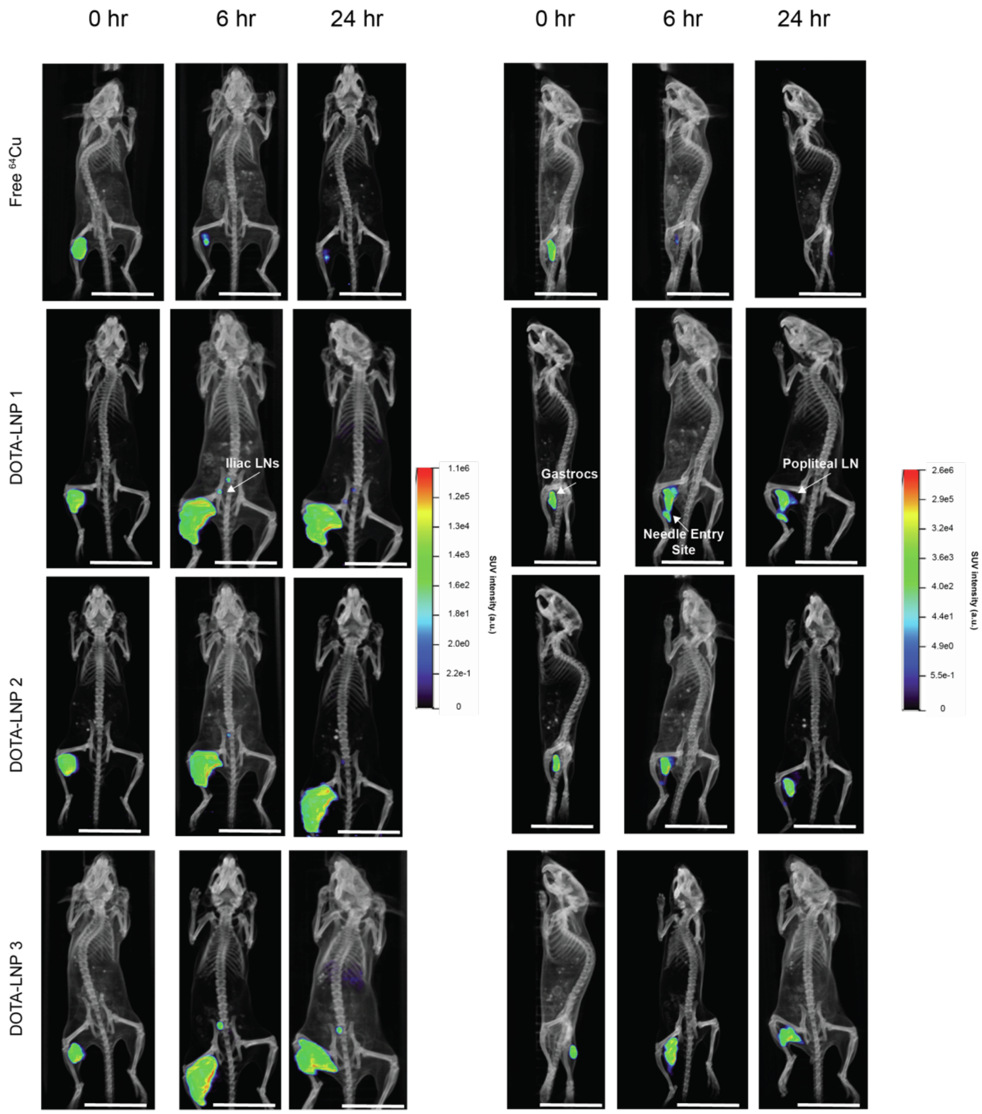

Figure S3. PET-CT projections for ^{64}Cu and remaining DOTA-LNP mice. Projections rendered in AMIDE software with intensity thresholds set the same across all groups/individual mice. Shown in the left panels and right panels are images of the same individual mice at the different timepoints, showing two different angles of view with two different intensity thresholds, to enable visualization of weak signal in the iliac LNs (left panels) and separately, brighter signals at the needle insertion site and draining popliteal LNs (right panels). Scale bars 50 mm.

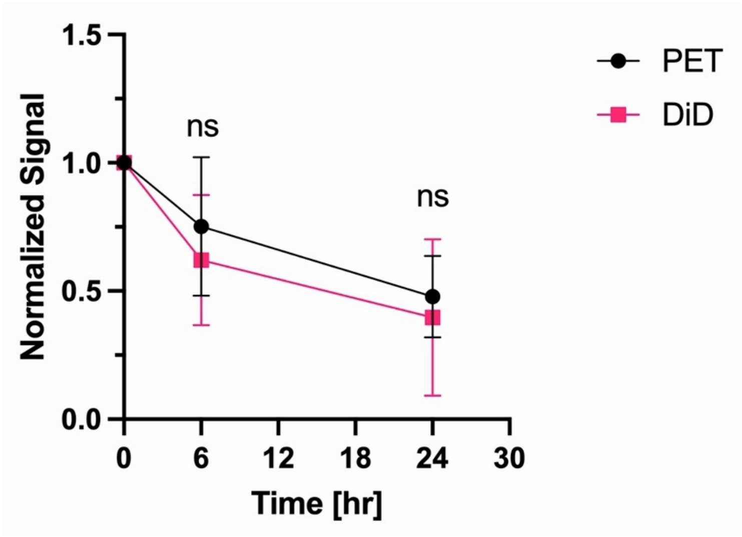

Figure S4. LNP signal clearance comparing lipid fluorescence tracer DiD vs. PET signal exhibit similar kinetics. PET/DiD tracer signals (normalized to the time zero initial signal) versus time in the injected muscle site. For both studies, LNPs were administered i.m. at a 10 µg dosage in the gastrocnemius muscle. Statistical significance was determined by two-way ANOVA followed by Tukey's post hoc test. *P < 0.05; **P < 0.01; ***P < 0.001; ****P < 0.0001. All data show means ± SEM.

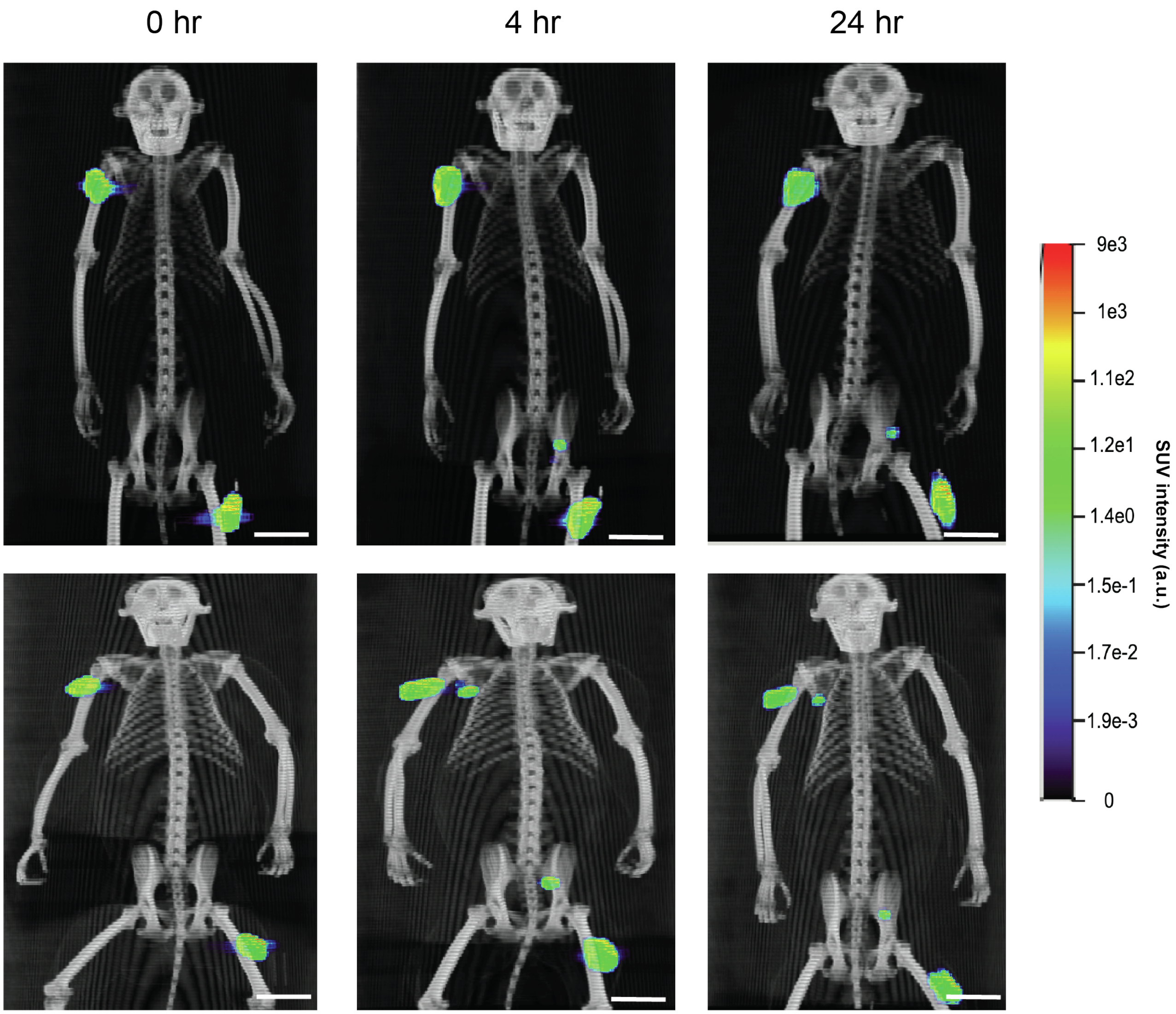

Figure S5. PET-CT projections for two-injection DOTA-LNP immunized NHPs. Projections rendered in AMIDE software with thresholds set constant across all animals. Scale bars 50 mm.

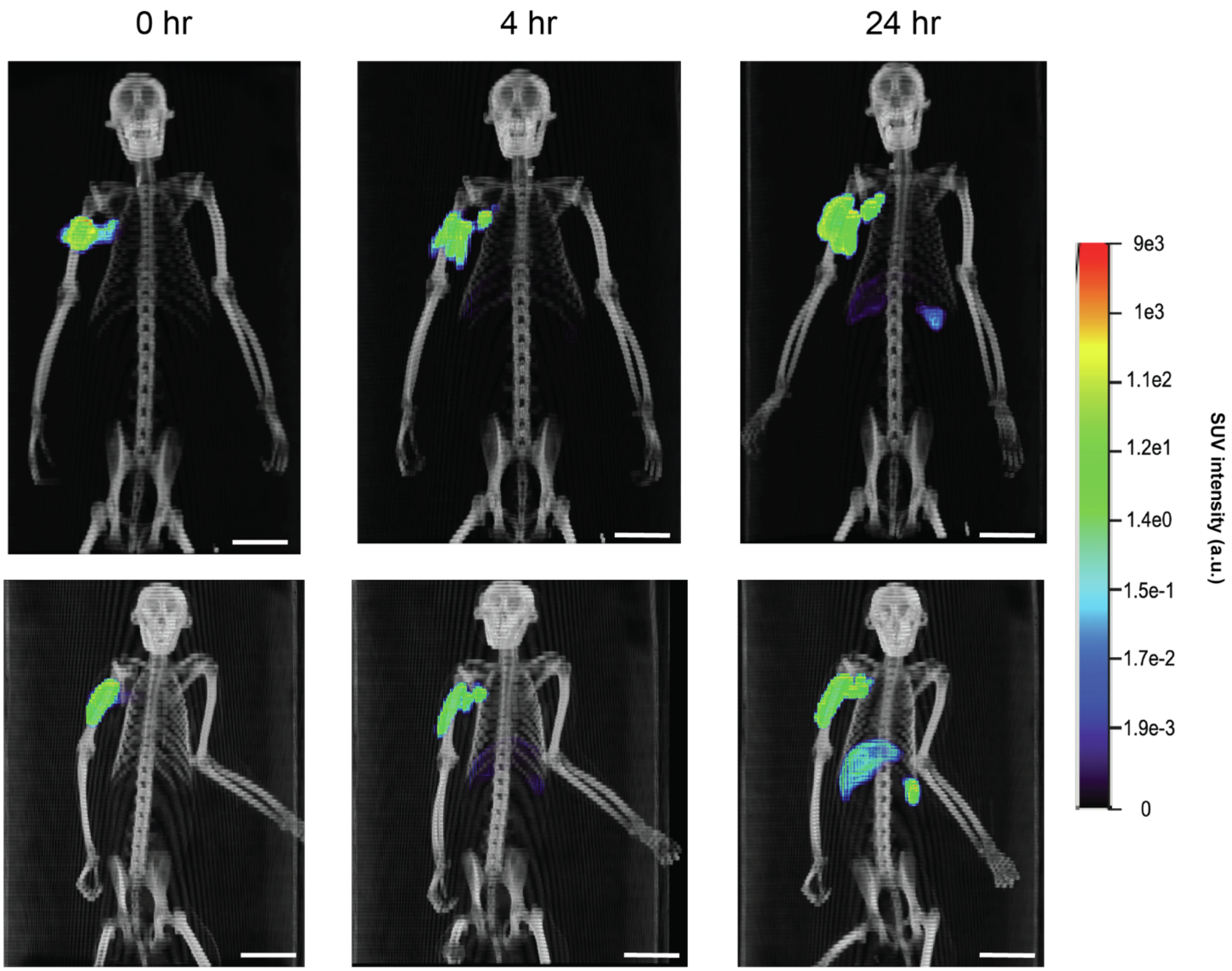

Figure S6. PET-CT projections for single-injection DOTA-LNP immunized NHPs. Projections rendered in AMIDE software with thresholds set constant across all animals. Scale bars 50 mm.

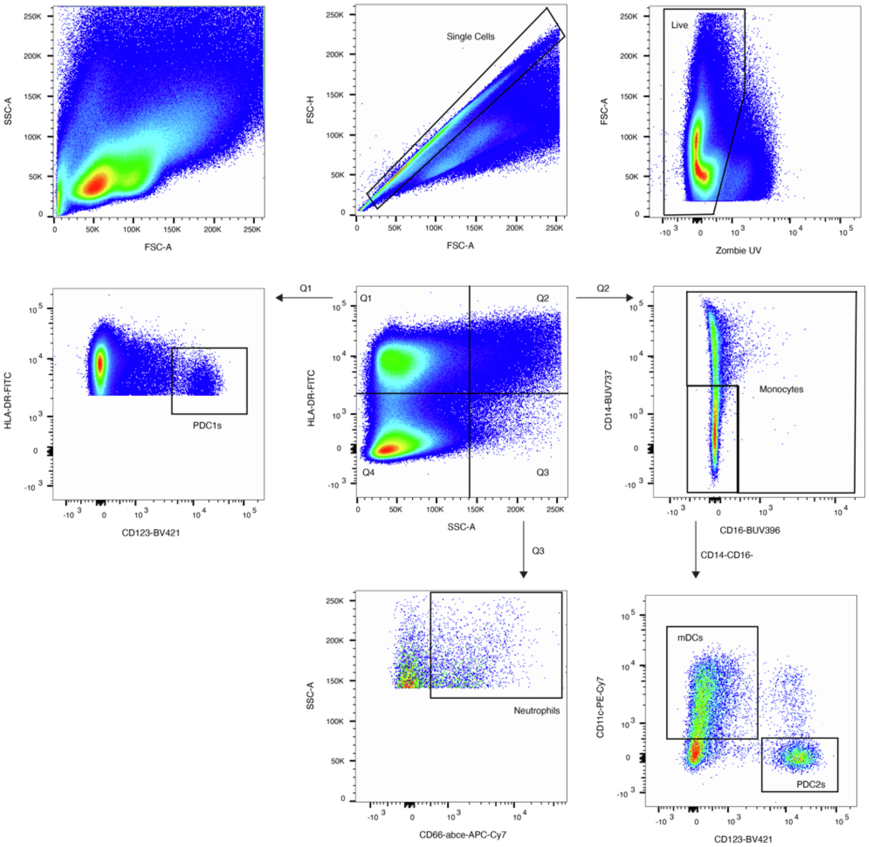

Figure S7. Flow cytometry gating strategy for NHP myeloid cell analysis.

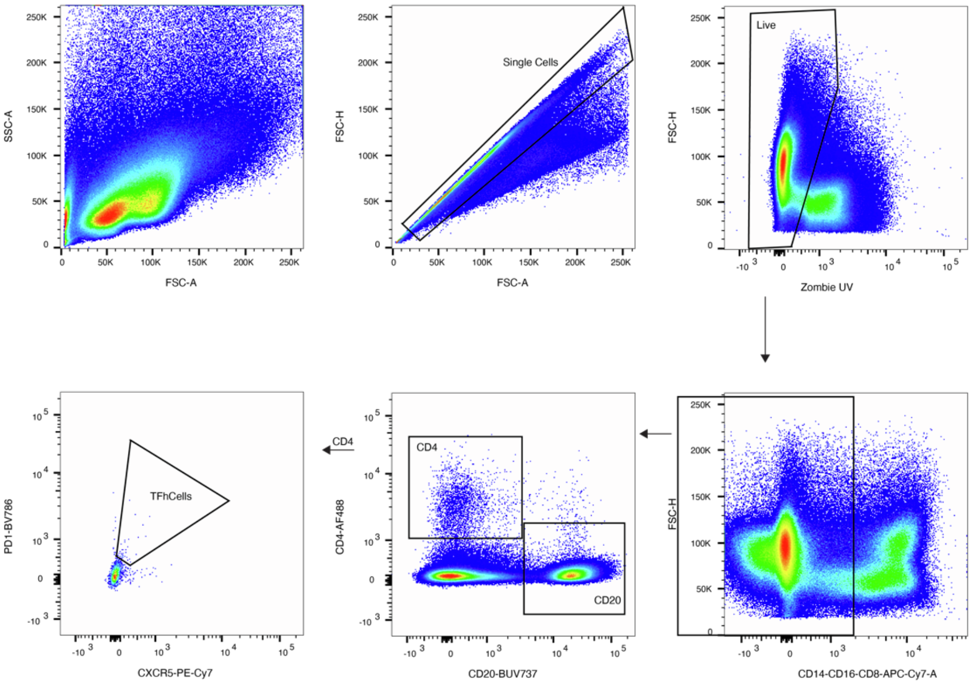

Figure S8. Flow cytometry gating strategy for NHP lymphocyte analyses.

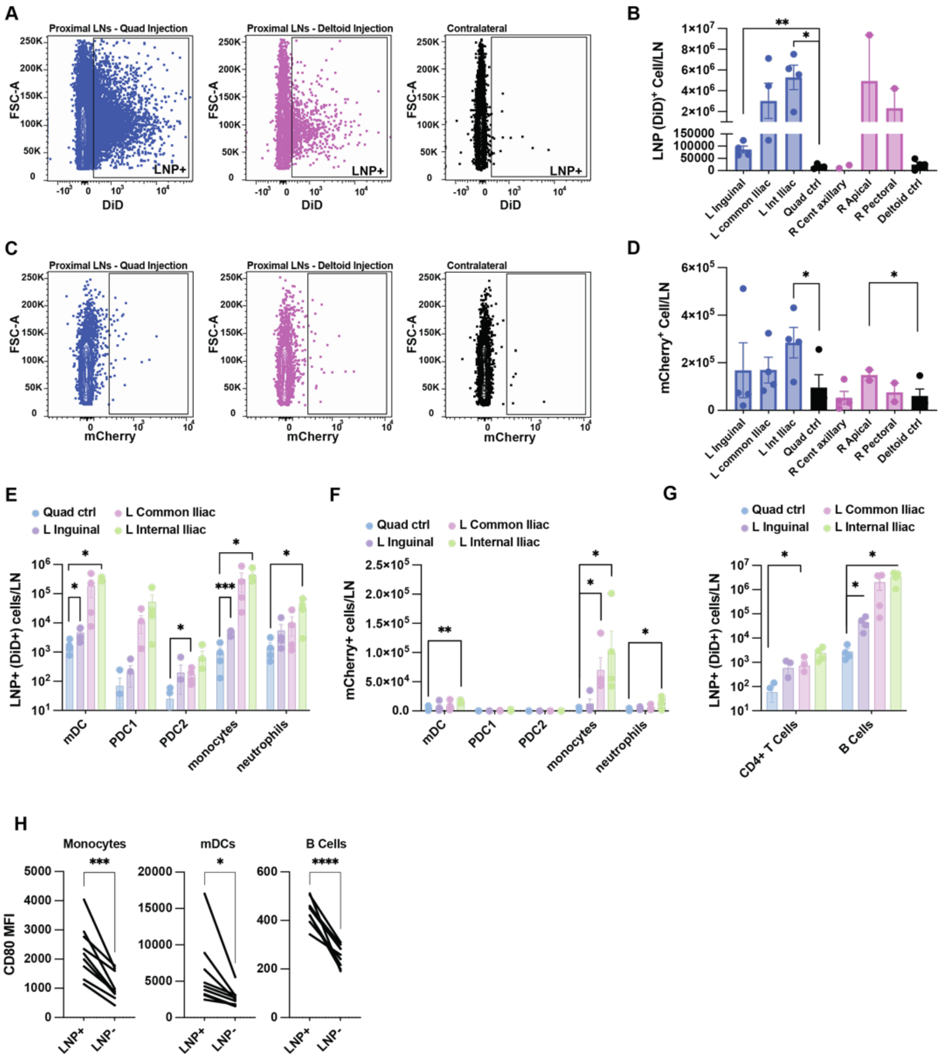

Figure S9. Monocytes and dendritic cells acquire LNPs in NHP draining LNs. (A) Representative flow cytometry contour plots of LNP (DiD) signal in all cells. **(B)** Total cell counts gated on LNP⁺ (DiD) signal. **(C)** Representative flow cytometry contour plots of mCherry signal. **(D)** Total cell counts gated on mCherry⁺ signal. **(E)** Percentage cells positive for LNP signal within specific myeloid populations in LNs draining the quadricep injection site. **(F)** Number of cells positive for mCherry⁺ signal within specific myeloid populations in LNs draining the quadricep injection site. **(G)** Percentage cells positive for

LNP signal within specific lymphoid populations in LNs draining the quadricep injection site. **(H)** Geometric mean fluorescent intensity of CD80-BV650 signal in LNP^+ relative to LNP^- cells within LNs with high LNP uptake (>1%). Cell populations were gated as follows: mDCs ($SSC^{high}CD14^-CD16^-$ $MHCII^+CD11c^+$); pDC1s ($SSC^{low}MHCII^+CD123^+$); pDC2s (SSC^{high}CD14-CD16-$MHCII^+CD11c^-CD123^+$); Monocytes ($SSC^{high}MHCII^+CD14^+$ and/or $CD16^+$); Neutrophils ($SSC^{high}MHCII^-CD66abce^+$); Lin^-($CD8^-CD14^-CD16^-$).

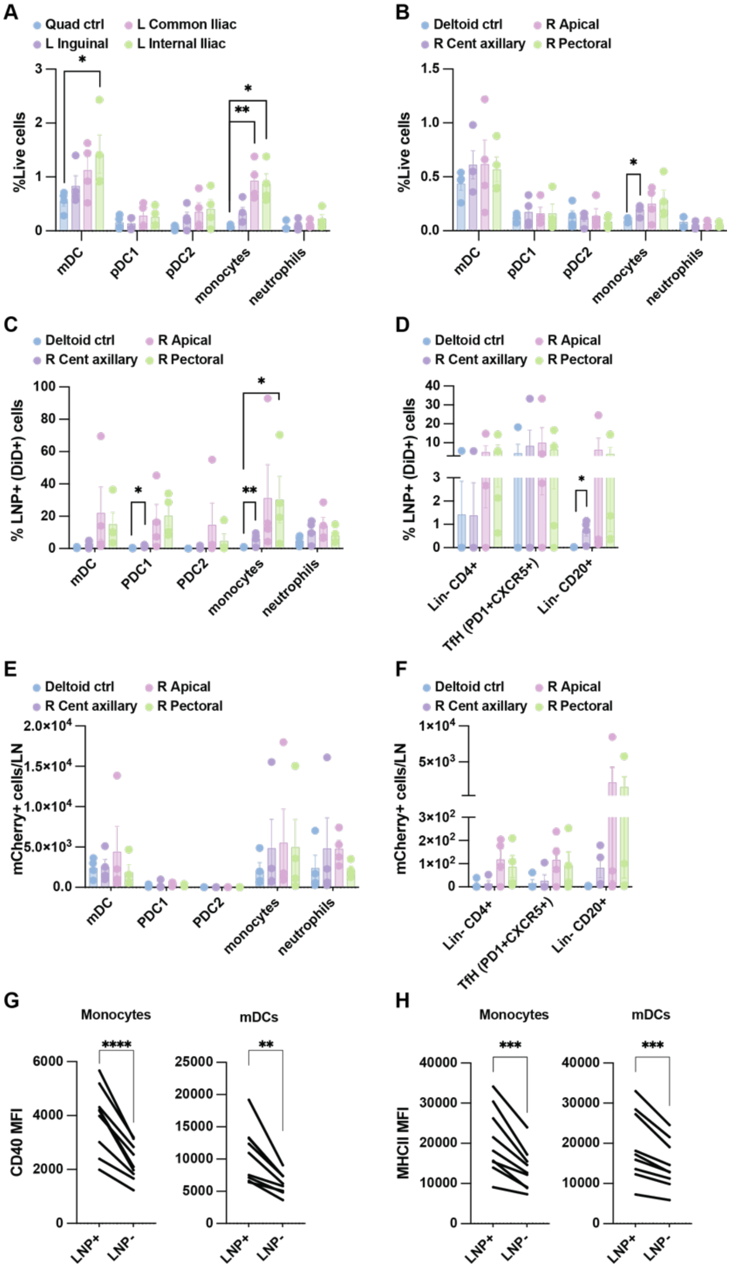

Figure S10. APCs found to be cell type responsible for LNP uptake and mRNA translation in draining LNs. (A) Myeloid infiltrate as % of live cells in LNs draining the quadricep injection site. **(B)** Myeloid infiltrate as % of live cells in LNs draining the deltoid injection site. **(C)** Percentage cells positive for LNP signal within specific myeloid

populations in LNs draining the deltoid injection site. **(D)** Percentage cells positive for LNP signal within specific lymphoid populations in LNs draining the deltoid injection site. **(E)** Number of cells positive for $mCherry^{+}$ signal within specific myeloid populations in LNs draining the deltoid injection site. **(F)** Number of cells positive for $mCherry^{+}$ signal within specific lymphoid populations in LNs draining the deltoid injection site. **(G)** Geometric mean fluorescent intensity of CD80-PerCP-Cy5.5 signal in LNP^{+} relative to LNP^{-} cells within LNs with high LNP uptake (>1%). **(H)** Geometric mean fluorescent intensity of MHCII-FITC signal in LNP^{+} relative to LNP^{-} cells within LNs with high LNP uptake (>1%). Cell populations were gated as follows: mDCs ($SSC^{high}CD14^{-}CD16^{-}$ $MHCII^{+}CD11c^{+}$); pDC1s ($SSC^{low}MHCII^{+}CD123^{+}$); pDC2s ($SSC^{high}$CD14-CD16-$MHCII^{+}CD11c^{-}CD123^{+}$); Monocytes ($SSC^{high}MHCII^{+}CD14^{+}and/orCD16^{+}$); Neutrophils ($SSC^{high}MHCII^{-}CD66abce^{+}$); Lin^{-}($CD8^{-}CD14^{-}CD16^{-}$

Supplemental Movie Titles

Video S1. Mouse PET-CT video projection for free ^{64}Cu control (from left to right) at 0, 3, and 24 hr timepoints.

Video S2. Mouse PET-CT video projection at 0 hr timepoint.

Video S3. Mouse PET-CT video projection at 24 hr timepoint.

Video S4. NHP mCherry study PET-CT video projection at 0 hr timepoint.

Video S5. NHP mCherry study PET-CT video projection at 4 hr timepoint.

Video S6. NHP mCherry study PET-CT video projection at 24 hr timepoint.

Video S7. NHP N332-GT2 study PET-CT video projection at 0 hr timepoint.

Video S8. NHP N332-GT2 study PET-CT video projection at 4 hr timepoint.

Video S9. NHP N332-GT2 study PET-CT video projection at 24 hr timepoint.
